# An estimate of burnout prevalence among oncology nurses

**DOI:** 10.1186/s12912-024-02421-x

**Published:** 2024-10-15

**Authors:** Madeleine Helaß, Imad Maatouk

**Affiliations:** 1 Department of General Internal Medicine and Psychosomatics, University Hospital Heidelberg, Im Neuenheimer Feld 410, 69120 Heidelberg, Germany; 2 Department of Internal Medicine II, Section of Psychosomatic Medicine, Psychotherapy and Psychooncology, Julius-Maximilian University Würzburg, Oberdürrbacher Str. 6, 97080 Würzburg, Germany

**Keywords:** Burnout, Oldenburger Burnout Inventory, Oncology nurse, Prevalence, Healthy work environment

## Abstract

**Background:**

Registered nurses (RNs) in oncology must cope with the suffering of patients, the inevitability of death and their own transience. This poses a possible risk for the development of burnout, which can result in low job satisfaction and ultimately an increased intention to leave the job. Our aim was to assess psychological distress in registered nurses working in oncology.

**Objective and method:**

A cross-sectional survey with the Oldenburger Burnout Inventory was presented to nurses within the German Cancer Society. It collected data on psychological distress via two subscales, exhaustion and disengagement. Socio-demographic data were assessed.

**Results:**

Among 83 participating nurses, we found a prevalence of high disengagement in 17 oncology nurses (20.48%) and high exhaustion (M_Exh_>2.5 = burnout) in 44 (53.00%). Looking at the highest values of both scales, 18.08% of respondents were at high risk for psychological distress. There was a low correlation between disengagement and age (*r* = 0.331, *p* < 0.01). The risk of high disengagement among nurses older than 50 (*n* = 9, 52.94%) was three times higher than for those who were 50 or younger (*n* = 8, 47.06%) (RR = 8.642, 95% CI: 1.475–5.749, *p* < 0,01).

**Conclusion:**

This survey highlights a high rate of burnout among German oncology nurses. Interventions should be developed, implemented, and delivered in an age-appropriate manner. To ensure high-quality care and patient safety, oncology nurses should be offered preventive mental healthcare services later in their careers.

**Clinical trial registration number:**

The study was registered with the German Clinical Trials Register (DRKS500018851).

**Supplementary Information:**

The online version contains supplementary material available at 10.1186/s12912-024-02421-x.

## Background

Psychological distress in the nursing profession is increasingly becoming a focus of research, and studies on the predisposing factors and consequences of psychological distress reveal unfavourable intraindividual as well as environmental factors that influence nurses’ mental health and quality of life.

Psychological distress in oncology is often operationalized through the concept of burnout, which is seen as ineffective coping with work-related stressors, measured with the Maslach Burnout Inventory (MBI) with the scale emotional exhaustion, depersonalization, and personal achievement [1]. Considering the criticisms of the MBI, such as psychometric weakness (i.e., factor validity, one-sided wording of items) (for further information see 2, 3), the results seem to distort the situation of nurses [2]. Based on the already known difficult situation of nurses in general, a more differentiated view of psychological stress in the medical field of oncology takes place in the context of demographic factors. Accordingly, there may be rather unclear implications as to how the deficits in support for nurses can be remedied. This study surveyed the prevalence of psychological distress among oncology nurses with the help of the Oldenburg Burnout Inventory (OLBI) to derive more specific implications for research and practice. The OLBI conceptualizes psychological distress through two dimensions: exhaustion and disengagement. The dimension exhaustion refers “to general feelings of emptiness, overtaxing from work, a strong need for rest, and a state of physical exhaustion” [3]. Exhaustion refers to the long-term consequence of intense physical, affective and cognitive stress. OLBI_dis_ refers to distancing oneself from one’s work and the attitudes associated with work and to negative, cynical attitudes and behaviours towards one’s work in general (ibid.). The last issue in particular seemed crucial for the survey of nurses, as work-related attitudes are predictors of job performance [4] and job involvement is related to job satisfaction [5].

Hospital nurses are exposed to various psychosocial and work-related stressors, such as shift work, patient and relative care, and new and higher demands, such as an increased workload with staff shortages [6, 7]. The care of people with cancer in oncology poses a particular challenge for all professional groups involved, with registered nurses (RNs) being of particular importance, as they are confronted with “patients’ death, delivering bad news, the limits of the treatment, and the worry about their own death” [8, 9] and may therefore be at higher risk of subsequent symptoms [10].

Health-threatening consequences such as burnout [11] occur more frequently among oncology nurses than among nurses in other medical fields [12]. In a meta-analysis, the prevalence of high emotional exhaustion and depersonalization among oncology nurses was 30% and 15%, respectively, and low personal accomplishment was 35% [8] and was thus higher than the high-burnout prevalence of 11% among nurses worldwide across all medical fields [13].

Although the results on the prevalence of burnout among oncology RNs are consistent, the empirical evidence offers mixed views on burnout intensity among them. Some reported low 29.6% [14] to moderate expression [15], and other empirical studies indicated that three-quarters of oncology nurses [16] are in the most severe stages of burnout. Due to the inconsistent findings on the intensity of burnout among oncology nurses on the one hand and the proven weaknesses of the MBI and the associated results on the prevalence of burnout on the other, the present study aimed to assess psychological distress in the context of relatively underexplored demographic factors in terms of probability of occurrence and severity using the OLBI.

## Methods

### Design

Oncology RNs from the German Cancer Society were asked via email from a mailing list of the Academy of Health Professionals to participate in an internet-based survey on psychological stress. Access to the survey was only possible with the link sent by email to interested participants. The survey, provided by SoSci Survey, was open for three months, April-July 2020, without any follow-up. Participation in the survey was voluntary and anonymous. Informed consent was obtained from participants.

The 16-item OLBI [3] measures two subscales, exhaustion and disengagement (8 items per subscale), on a 4-point Likert scale (1 = “strongly disagree” to 4 = “strongly agree”). Four items of each subscale were inverted, so that the scaling was also inverted during the evaluation. Both subscales had a good reliability of .85 [3]. Bivariate correlations between the two subscales were 0.55 (*p* < 0.01) for health care workers with mean levels of exhaustion (M = 2.53) and disengagement (M = 2.38) [3]. Since there is no standard cut-off for burnout in OLBI, we first used M_OLBI_ ≥ 2.18 as the mean value of both disengagement and exhaustion scales [17]. Further analyses are limited to the more sensitive cut-off of the exhaustion subscale M_Exh_ ≥ 2.5 [18] as the ‘burnout measure’.

We assessed socio-demographic data, medical field, professional experience, place of work, employment status, work in the inpatient palliative care unit (PCU) as well as participation in the outpatient palliative care team (*Spezialisierte Ambulante Palliativversorgung* (SAPV)), including the estimated proportion of total working time, proportion of inpatient work, work time with tumour patients and the estimated proportion of work time with palliative patients. Sample size was not pre-calculated, as this is an exploratory study.

### Data analysis

Absolute and relative frequencies were calculated with categorial data, such as gender, status of employment, medical field, working in PCU and in SAPV, and professional experience. For continuous variables, we present the mean, standard deviation and range. One participant whose data was incomplete was nevertheless included, as the missing data only included socio-demographic data (SAPV-activity). First, we used one-way analysis of variance (ANOVA) as an alternative to the t test to examine differences in the subgroups of sociodemographic variables in exhaustion, disengagement, and burnout. Second, correlation coefficients were examined among all study variables. Third, multiple regression analyses were performed between disengagement and exhaustion as dependent variables and the sociodemographic factors as independent variables to examine possible predictors for the OLBI measures. The risk ratio (RR) was used to determine the risk of burnout. For all tests, *p* < 0.05 was considered to indicate statistical significance. For subgroup analyses, interval-scaled measures were divided, using quartiles as cut-offs (P_25_, P_50_, P_75_). The Pearson’s chi-square test was used to analyse the correlation of categorical data. Cramer`s-V was used to measure the strength of the association of two nominal data (subgroup analysis). Analysis was performed using IBM SPSS Statistics 27.

## Results

### Characteristics of participants

Of a total of approximately 226 oncology RNs, 83 participated, for a response rate of 36.73%. The mean age was 40.30 (SD = 10.74, range 23–62). The sample was predominantly female (*n* = 69, 83.13%). Professional experience varied widely between 2 and 49 years (median = 15–19 years (8.07%, IQR = 10–25 years). All nursing participants were salaried in a hospital (*n* = 83, 100%), mainly in haematology and oncology (*n* = 47, 56.63%). The participants worked on average 89.93% (SD = 17.66, range 6-100) of their time with tumour patients. *N* = 6 (7,23%) worked within a palliative care unit, spending 58.17% (SD = 34.98, range 16–100) of their work time there. One participant (1.2%) was part of an outpatient palliative care team (*spezialisierte ambulante Palliativversorgung*, SAPV), which accounted for 100% of their work time on average. Absolute and relative frequencies are shown in Table 1. Mean values, standard deviations, ranges and correlation coefficients can be found in Table 2. Internal consistency was moderate (Cronbach`s α = 0.600).

**Table 1:** Descriptive Statistics of Sociodemographic Factors in disengagement and exhaustion

		Disengagement									Exhaustion										
		Low^a^		Medium		High					Low		High (Burnout)^b^					Total			
		N	%	N	%	N	%				N	%	N	%				N	%		
		23	27.71	43	51.80	17	20.48				39	47.00	44	53.00				83	100		
Sex	Male	4	28.57	7	50.00	3	21.43				9	64.90	5	35.71				14	16.87		
	Female	23	33.33	32	46.38	14	20.29				30	43.35	39	56.65				69	83.13		
Employment status	Employed in hospital	23	27.71	43	51.80	17	26.48				39	47.00	44	53.00				83	100		
Medical field	Internal medicine haematology/oncology	12	25.53	23	48.94	12	25.53				20	42.55	27	57.45				47	56.63		
	Gastroenterology	0	0	0	0	0	0				0	0	0	0				0	0		
	Pneumology	0	0	1	50.00	1	50.00				0	0	2	100				2	2.40		
	Gynaecology	1	16.67	4	66.67	1	16.67				1	16.67	5	83.33				6	7.22		
	Radiation therapy	2	66.67	0	0	1	33.33				1	33.33	2	66.67				3	3.61		
	Neurology	0	0	2	100	0	0				0	0	2	100				2	2.41		
	Surgery	1	20.00	3	60.00	1	20.00				4	80.00	1	20.00				5	6.02		
	Other	0	0	17	20.48	0	0				13	72.22	5	27.77				18	21.69		
	Not specified	0	0	0	0	0	0				0	00	0	0				0	0		
PCU	Yes	3	50.00	3	50.00	0	0				5	83.33	1	16.67				6	7.23		
	No	20	25.97	40	51.95	17	22.08				34	50.75	43	49.25				77	92.77		
	Not specified	0	0	0	0	0	0				0	0	0	0				0	0		
SAPV	Yes	1	100	0	0	0	0				1	100	0	0				1	1.2		
	No	22	27.16	5	6.17	64	79.01				38	46.91	43	53.09				81	97 − 9		
	Not specified	0	0	1	100	0	0				0	0	0	0				1	1.20		
Professional experience	< 2	1	100	0	0	0	0				0	0	1	100				1	1.20		
	2–4	2	40	2	40	1	20.00				4	80.00	1	20.00				5	6.02		
	5–9	7	33.34	11	52.38	4	15.39				12	57.14	9	42.86				21	25.30		
	10–14	2	20.00	7	70.00	10	10.0				2	20.00	8	80.00				10	12.00		
	15–19	3	20.00	9	60.00	3	20.00				6	40.00	9	60.00				15	8.07		
	20–24	5	35.71	7	50.00	2	14.29				7	50.00	7	50.00				14	16.87		
	25–29	1	20.00	3	60.00	2	40.00				0	0	5	100				5	6.02		
	30–34	2	33.33	2	33.33	2	33.33				4	66.67	2	33.33				6	7.22		
	35–39	0	0	1	33.33	2	66.67				3	66.67	1	33.34				3	3.61		
	40–44	0	0	1	50.00	1	50.00				1	50.00	1	50.00				2	2.41		
	> 45	0	0	1	100	0	0				1	100	0	0				1	1.20		

^a^ low (< 1.63), medium (1.63–2.24), high (> 2.24); ^b^ Cut-Off = 2,5

**Table 2:** Mean, standard deviations, ranges and correlation coefficients of sociodemographic data and OLBI measures of oncology RNs

No.	Item	*N*	M	SD	range	1	2	3	4	5	6	7	8	9	10	11	12	13
1	Age	83	40.30	10.744	23–62	1												
2	Gender	83				0.237	1											
3	Professional experience	83	15–19^3^			**.677** ^a^	.**486**^b^	1										
4	Medical field	83				0.008	0.186	0.336	1									
5	Work time with tumour patients %	82	89.93	17.662	6-100	0.028	**− .285** ^a^	**− .0**84	**− .263** ^b^	1								
6	Palliative tumour patients %	83	45.25	27.628	1-100	0.097	0.030	0.057	0.003	0.217	1							
7	Inpatient work %	83	71.05	40.604	1-100	**− .325** ^a^	**.223** ^**b**^	− 0.097	**.239** ^b^	− 0.580	0.140	1						
8	Working on a palliative care unit	6				0.603	0.001	0.342	0.164	0.628	0.712	0.591	1					
9	- Palliative care %	6	58.17	34.982	16–100	0.603	− 0.591	0.692	− 0.269	0.628	0.712	0.591	0.000	1				
10	Exhaustion	83	2.51	0.620	1.13–3.88	0.118	− 0.159	0.093	− 0.198	− 0.049	0.029	− 0.121	0.138	− 0.256	1			
11	Disengagement	83	1.89	0.469	1.00–3.38	.**331**^a^	0.066	0.140	0.016	0.016	0.022	− 0.163	0.207	− 0.165	.**600**^a^	1		
12	Exhaustion > 2.5 (Burnout)	44				− 0.067	0.156	0.413	0.398	0.009	− 0.019	0.085	0.203	0.100	**.826** ^a^	**0.459** ^**^	1	
13	Disengagement > 2.24	17				0.262	0.011	0.326	0.249	0.129	0.001	− 0.121	0.142	− 0.165	.**499**^a^	0.756**	0.358**	**1**

^a^ Correlation is significant at a level of *p* < 0.01 (2-tailed); ^b^ Correlation is significant at a level of *p* < 0.05 (2-tailed). M, Mean; SD, standard deviation; ^3^median

### Disengagement, exhaustion, and burnout

The mean value on the disengagement scale was 1.89 (SD = 0.469, range 1.00-3.38). Correlation analyses revealed a significantly low correlation between disengagement and age (*r* = 0.331, *p* < 0.01), which means that the older the RN is, the higher the disengagement. Using mean age as a cut-off, the t test showed a significant difference (t(81) = 2.758, *p* < 0.01) between nurses younger than 40 years (*n* = 44, M = 1.759, SD = 0.389) and older than 40 years (*n* = 39, M = 2.032, SD = 0.511), indicating a statistically significant increase in disengagement beginning at age of 40.

To evaluate the scale of disengagement, the range of values was divided into three parts and designated as low (< 1.63), medium (1.63–2.24) and high (> 2.24). *N* = 23 (27.71%) showed low disengagement (M = 1.40, SD = 0.183, range 1-1.5), *n* = 43 (51.80%) showed medium disengagement (M = 1.90, SD = 0.151, range 1.63–2.13) and *n* = 17 (20.48%) showed high disengagement (M = 2.58, SD = 0.336, range 2.25–3.38). High disengagement was mainly found among women (*n* = 14, 20.29%), non-PCU workers (*n* = 17, 22.08%), and non-SAPV workers (*n* = 64, 79.01%). Using 50 years as a cut-off at the highest quartile (P_75_), Pearson’s chi-square test confirmed an association between age greater than 50 years and high disengagement: χ^2^ [1] = 8.642, *p* < 0.01, φ = 0.323. The risk of high disengagement among nurses older than 50 (*n* = 9, 52.94%) was three times higher than for those who were at least 50 (*n* = 8, 47.06%) (RR = 2.912, 95% CI: 1.475–5.749, *p* < 0,01). The analysis of the exhaustion scale showed no significant results with sociodemographic data.

The correlation coefficient (Cramer`s-V) showed a significantly low correlation between gender and burnout (*r* = 0.156, *p* < 0.01). The t test was not significant.

Further analyses showed a significantly low positive correlation between high disengagement and burnout (Cramer`s-V = 0.358, *p* = 0.01). When the highest severity levels of both OLBI scales were considered together, *n* = 15 (18.08%) showed the most severe psychological stress. The vast majority of respondents (*n* = 34, 77.27%) with burnout had low and medium disengagement scores (Table 3). The regression analyses did not reveal any significant correlations, neither for the overall model nor for the individual factors.

**Table 3:** Frequency distribution of disengagement and exhaustion severity levels

		Disengagement								
		low		medium		high		total		
		N	%	N	%	N	%	N	%	
Exhaustion	low	16	19.28	21	25.30	2	2.41	39	46.99	
	high	7	8.43	27	23.53	15	18.07	44	53.01	
	total	23	27.71	38	45.78	17	20.48	83	100	

## Discussion

The aim of the present study was to evaluate exhaustion, disengagement, and burnout among oncology RNs in the context of sociodemographic factors. *N* = 17 (20.48%) nurses showed an increased value on the disengagement scale. In addition, we found that disengagement correlates slightly positively with age and that nurses older than 50 years are at threefold increased risk for high disengagement. Examining the prevalence of burnout, we found 53.00–71.08%, depending on which cut-off was used. If we consider the maximum groups of disengagement and exhaustion, we found a prevalence of 18.08% (*n* = 15) as a high-burdened group for psychological stress.

In our study, oncology nurses showed increased scores on the disengagement scale, *n* = 17 (20.48%) cases. To date, there have been no studies that allow a comparison of the OLBI_dis_ scale with RNs or oncology RNs. Since OLBI_dis_ is similar to MBI_DP_ [19], a comparison with the high-risk prevalences of the MBI_DP_ scales [20] was used for classification. In a recent systematic review, MBI_DP_ ranged from 8.6 to 29.8% [21]. High disengagement can have an impact on the nurse-patient relationship and thus affect the quality of care, as well as the oncological patients perception of pain, confidence, hope and thus also the course of the disease [22].As Mukherjee and Tennant [2] already pointed out, one should be cautious when interpreting the depersonalization scale of the MBI due to the questionable interval levels and “floor effect”, and our comparison should also be interpreted with caution.

Our study showed that OLBI_dis_ was positively related to age, confirming previous results on work-related stress in outpatient oncology nurses [23, 24]. The threefold increased risk for high disengagement after the age of 50 found in our study contradicts previous findings, in that younger oncology employees in particular achieved higher scores [25]. This contradiction can be explained as follows. The inner distancing from the patient’s suffering can be understood as a consequence of the special demands of oncology nursing and therefore as a problem-solving attempt to regulate unpleasant emotions over time to protect mental health [26].

It is worth noting that high disengagement in our study was predominantly found among nurses who were not involved in palliative care and confirms the observation that palliative care units revealed significantly less emotional exhaustion and depersonalization [27]. Gama, Barbosa, and Vierira come to the conclusion that meaning and purpose in life and attitude towards death are protective factors. Consequently, training measures, supervision, an education that are suitable for palliative care should also be offered to oncological staff.

The prevalence for burnout in our study was 71.08% (*n* = 59, with M_OLBI_-cut-off = 2.18) and 53.00% (*n* = 44, M_exh_-cut-off = 2.5), far above the average burnout rate of nurses worldwide and across all medical fields (11.23%, 14), especially of oncology RNs (20%) [8], although we used the stricter cut-off. Focusing on the highest values of both OLBI scales, 18.07% of our respondents were at high risk for mental stress. In a systematic review, significantly lower burnout scores of oncology RNs have been recorded to date: those using the MBI scales ranged between 21 and 32% depending on the scale. Europe and Asia had the highest depersonalisation scores and Asia and Canada the lowest personal accomplishment scores [28]. A comparable study from 2022 showed similarly high prevalence among oncology nurses at 46% [29] A reason for this may be that the studies took place during the COVID-19 pandemic, and there has been evidence of increased nursing burnout in various medical fields [30–32], especially for non-frontline nurses [33], where nurses had to deal with uncertainty, fear and increased staff shortages as a result of quarantine and delegations to COVID-19 care units [21]. On the other hand, this can be seen as a result of the worsening of the precarious situation in nursing.

Three-quarters of the participants succeeded in maintaining a low to medium level of disengagement despite burnout symptoms. This points to the particular strength of the staff in the field who, despite exhaustion, had managed not to completely distance themselves from their work and patients and were able to stay connected to them. This could reflect why nurses in oncology still perceive the work with patients with tumour diseases as valuable and enriching.

### Strengths and limitations

This is the first study examining two dimensions of burnout in a German sample of oncology nurses and shows a higher prevalence among oncology nurses than in other medical disciplines. With a response rate of 36%, we could reach a large group with a wide range of professional experience. However, this survey has several limitations. As this is an exploratory study, various sample biases could not be avoided but must be taken into account, e.g., distortions could occur due to a lack of randomisation, as only the most severely affected nurses may have reported (participation bias) or participants may have taken part in the study with different personal intentions (volunteer bias).,Furthermore, the number of oncology nurses who responded was limited; oncology nurses with a higher symptom load might have reported at a higher frequency, leading to an overestimation of the prevalence of burnout and risk of high disengagement. It should be mentioned that outliers and extreme values of participants can distort the value due to mean value cut-offs. Nevertheless, we decided in favour of its use, as this makes comparisons with other study populations calculable. Then, additional stressors associated with coronavirus disease 2019 (COVID-19) like social distancing [34] might have influenced the results of this survey, as social support is a crucial resource for dealing with work-related stress. Longitudinal or cohort studies, and follow-ups of already conducted prevalence studies are necessary to verify this causal eventuality. As a last point, this study is related to its cross-sectional design and does not allow either temporal or causal inferences. Further investigations with consecutive measurements are required to obtain a more detailed understanding of burnout among oncology nurses.

## Conclusion

This survey highlights a high rate of burnout among German oncology nurses, with a focus on professional burden during the later years of their careers. Using a diagnostically sufficient instrument, the OLBI with the scales exhaustion and disengagement, interventions for the reduction of psychological stress of older nurses in oncology can be developed more specifically. Since age has emerged as a key determinant of disengagement, interventions should be developed, implemented, and delivered in an age-appropriate manner.

Effective training measures to reduce burnout in oncology nurses that focus on communication, self-care, relaxation, and cognitive behavioural therapy measures have already been investigated in experimental studies [35, 36] Especially for oncology RNs, interventions to promote death self-efficacy, attitude towards death, and attitudes toward meaning and purpose in life and should be integrated into the package of postgradual training measures, as this correlates with burnout [37]. However, little attention has so far been paid to disengagement. Due to the conceptual similarity with Compassion Fatigue, which describes similarly high prevalence for burnout of oncology nurses [38], training measures should also include the promotion of emotion regulation and compassion satisfaction. Initial studies have already shown that preventive measures, which were effective in the prevention of burnout, also contribute to the reduction of compassion fatigue and maybe in disengagement [39]. As disengagement significantly affected changes in ITL over time [40, 41], nursing managers and colleagues should be alert to signs of disengagement when working with oncology staff over the age of 50 and offer interventions like short contacts or supervision. In addition to personal factors, organisational factors should also be taken into account, especially in the treatment of burnout in Europe [42]. Suitable measures can be offered at the level of work organisation, for example through flexible schedules, management level and career opportunities [43].

More support is necessary to mitigate potential stressors for oncology nurses within the healthcare system. To ensure high-quality care and patient safety, oncology nurses should be offered preventive mental healthcare services later in their careers.

## Electronic supplementary material

Below is the link to the electronic supplementary material.

Supplementary Material 1

## Data Availability

The datasets used and analysed during the current study are available from the corresponding author on reasonable request.
